# Editorial: Impact of environment, physical activity, nutrition and mental health in pediatric rheumatology diseases: towards an integrative approach in patient management

**DOI:** 10.3389/fnut.2023.1346589

**Published:** 2023-12-20

**Authors:** Alain Lefèvre-Utile, Etienne Merlin, Raphael Kraus, Jean Jacques De Bruycker

**Affiliations:** ^1^Department of General Pediatrics, Centre Hospitalier Universitaire Vaudois (CHUV), Faculté de Biologie et de Médecine (FBM), de l'Université de Lausanne (UNIL), Lausanne, Switzerland; ^2^Department of Pediatrics, Clermont-Ferrand University Hospital, Clermont-Ferrand, France; ^3^INSERM, CIC 1405, CRECHE Unit, Clermont Auvergne University, Clermont-Ferrand, France; ^4^Division of Pediatric Rheumatology and Immunology, CHU Sainte-Justine, Université de Montréal, Montreal, QC, Canada

**Keywords:** nutrition, sport, pediatric rheumatic disease, integrative medicine, non pharmaceutical intervention

## Introduction

Pediatric rheumatic diseases include various immune-mediated conditions such as inflammatory arthritides, vasculitides, autoinflammatory syndromes, and other autoimmune diseases. Although recent therapeutic advances (namely the introduction and continued development of biologic and small molecule drugs) have dramatically improved disease outcomes (increased likelihood of disease remission, decreased corticosteroid burden) and, subsequently, patient quality of life, a subset of children with rheumatic disease will nonetheless not experience satisfactory response to these therapies.

Relevant to therapeutic responders and non-responders alike, the negative influence of a sedentary lifestyle leading to immune dysregulation has been described. Therefore, exercise-driven non-pharmacologic interventions represent a promising new therapeutic direction. Our research describes the effects of such complementary, non-pharmacologic interventions on treatment response rate, remission rate, and quality of life.

## Evidence-based prescription of physical activity in juvenile idiopathic arthritis, review and perspective

Juvenile idiopathic arthritis (JIA) is the most common rheumatic disease in children. Children with JIA find themselves in a vicious cycle: joint pain results in decreased physical activity, which in turn may exacerbate disease activity and lead to unfavorable health outcomes. Over the past decades, there has been growing interest in the health benefits of increased overall physical activity as well as exercise interventions in young people with JIA.

Rochette et al. describe available data in support of prescribed physical activity/exercise in JIA as a behavioral, non-pharmacologic complement to medicinal therapies to attenuate inflammation and therefore disease symptoms and improve metabolism, sleep quality (including synchronization of circadian rhythm), mental health, and quality of life. They outline the current knowledge gaps, resultant clinical implications of available data, as well as necessary next steps for high-quality research. However, the current evidence base is lacking: rheumatologists at the bedside today are not equipped with adequate data nor professional organization recommendations to guide clinical practice.

Py et al. describe the ATHLETIQUE trial, which aims to evaluate the impact of a program integrating adapted physical activity (APA) disease activity in patients with JIA. A randomized, multicenter, open-label, controlled clinical trial, ATHLETIQUE will compare children aged 6 to 17 years who participate in an APA program for 3 months (in addition to use of a pedometer for 1 year) to controls. Measured outcomes include: disease activity (primary objective), fatigue, pain, quality of life, level of physical activity, functional capacities, and measured muscle strength. They hypothesize that positive results would enable a larger clinical research program allowing for further assessment and optimization of an APA protocol for children with JIA.

## Influence of dietary habits on biologic treatment response

The influence of diet on disease course in arthritis, putatively via immune dysregulation, is poorly studied in children. However, Overgaard et al. carried out a multicentre prospective cohort study of adult patients with chronic inflammatory disease demonstrated that patients with rheumatoid arthritis receiving a diet high in fiber and low in red and processed meats were significantly more likely to have favorable clinical response to medical therapies compared to patients with low fiber/high red and processed meat intake (82 vs. 35%; OR: 9.84, 1.35–71.56). No difference was found in other inflammatory conditions. These results support that the impact of nutrition on health in inflammatory disease must also be studied in children.

In this ongoing Research Topic, we aim to elucidate the influence of environment and non-pharmacologic therapeutic strategies on disease course in children with chronic immune-mediated diseases.

## Author contributions

AL-U: Conceptualization, Writing—original draft, Writing—review & editing. EM: Conceptualization, Writing—review & editing. RK: Methodology, Validation, Writing—review & editing. JD: Validation, Writing—review & editing.

